# Longitudinal Analysis of Paraspinal Muscle Cross-Sectional Area During Early Adulthood – A 10-Year Follow-Up MRI Study

**DOI:** 10.1038/s41598-019-56186-4

**Published:** 2019-12-20

**Authors:** Teemu Mäki, Petteri Oura, Markus Paananen, Jaakko Niinimäki, Jaro Karppinen, Juho-Antti Junno

**Affiliations:** 1 Research Unit of Medical Imaging, Physics and Technology, Faculty of Medicine, University of Oulu, Oulu, Finland; 2 Center for Life Course Health Research, Faculty of Medicine, University of Oulu, Oulu, Finland; 3 Medical Research Center Oulu, Oulu University Hospital and University of Oulu, Oulu, Finland; 4 Finnish Institute of Occupational Health, Oulu, Finland; 5 Department of Radiology, Oulu University Hospital, Oulu, Finland; 6 Cancer and Translational Medicine Research Unit, Faculty of Medicine, University of Oulu, Oulu, Finland; 7 Department of Anatomy, Faculty of Medicine, University of Oulu, Oulu, Finland; 8 Department of Archaeology, Faculty of Humanities, University of Oulu, Oulu, Finland

**Keywords:** Skeletal muscle, Epidemiology

## Abstract

Only a few previous studies have investigated paraspinal musculature (i.e., multifidus (MF), psoas major (PSM), erector spinae (ES)) in longitudinal, population-based settings. This study aimed to evaluate changes in the cross-sectional area (CSA) of the paraspinal muscles between the ages of 20 and 30 years. The study population consisted of a sub-cohort from the Northern Finland Birth Cohort 1986 (n = 298; 156 men, 142 women). Baseline magnetic resonance imaging was performed at a mean age of 21.3 years and follow-up imaging at 30.6 years. The CSA measurements were performed by tracing the paraspinal muscle outlines individually (MF, ES, PM) and all combined (total muscle area (TMA)) at the L4 cranial endplate level. The longitudinal data analysis was performed using generalized estimating equations modelling. The CSA of MF and ES increased during the follow-up among both sexes (men: MF + 5.7%, p < 0.001; ES + 2.7%, p = 0.001; and women: MF + 10.5%, p < 0.001; ES 9.2%, p = 0.001). The CSA of PM decreased among men (PM −4.0%, p < 0.001) but not among women (PM + 0.5%, p = 0.553). TMA increased significantly only among women (men: +0.5%, p = 0.425; women: +6.5%, p < 0.001). The increases in ES and TMA were more distinct among women than men (p < 0.001). Our study demonstrated clear age- and sex-related changes in paraspinal muscle size in early adulthood.

## Introduction

Recent studies have suggested that a small cross-sectional area (CSA) of paraspinal muscles predicts low back pain and disability^1,2^. Age-related structural changes in the lumbar spine, including CSA, morphology and composition, have been widely examined in cross sectional studies^1–7^, but longitudinal studies in this area are scarce^1,8,9^. The need for long-term cohort studies on this matter has been addressed^1^. Previous studies have shown that muscle mass decreases with age^10,11^. The CSA of the paraspinal muscles also tends to decrease with age, especially among the elderly, and lumbar muscle CSA is larger among men than women^7,12^. It is still unclear what happens to lumbar paraspinal musculature in early adulthood, as this has been rarely studied in longitudinal population-based settings.

The paraspinal musculature comprises muscle groups adjacent to the vertebrae and is responsible for the movement and stabilization of the spine. In the lumbar spine, these muscles include the multifidus (MF), erector spinae (ES), interspinales, intertransversarii, psoas major (PM) and quadratus lumborum^13,14^. The spinotransverse muscle group between the spinous and mammillary processes in the lumbar spine is referred to as the lumbar multifidus^15–17^. Its main function is to stabilize the spine^13^. The erector spinae muscle group extends along the whole length of the spine^17^. The function of these mucles is to extend the vertebral column^14^. The psoas major originates from the transverse processes of T12-L5 and inserts on the lesser trochanter of the femur^14,18,19^. Its main function is the flexion of the hip and it is considered the stabilizer of the lumbar spine^20^.

Previous studies have clearly indicated that the vertebral bone dimensions in the lumbar spine seem to increase between 20 and 30 years of age among both sexes^21^. Like those of bone, the dynamics of muscle mass have a multifactorial basis^22^. There are some indications that peak bone and muscle mass are achieved at about the same time^11^. Peak muscle mass seems to be attained around the third decade, after which a 3%–8% decrease per decade is estimated to occur^23^. However, a noticeable decrease in absolute muscle mass does not seem to take place before the end of the fifth decade in healthy individuals^23–25^.

The purpose of this study was to assess the change in paraspinal muscle CSA during a 10-year follow-up period in early adulthood between the ages of 20 and 30 years. We evaluated the changes in three muscle groups: MF, ES and PM. We hypothesized that the CSA of all muscle groups would slightly increase in the same way as vertebral dimensions.

## Material and Methods

### Study population

The study population consisted of a sample of the Northern Finland Birth Cohort 1985–1986 (NFBC1986)^26^, which comprises a total of 9479 Northern Finnish people with expected dates of birth between 1^st^ July 1985 and 30^th^ June 1986. Initially this cohort covered up to 99% of the infants in the area. The cohort is still followed periodically.

In 2001–2002, all cohort members with available addresses (n = 9215) were invited to fill in a questionnaire on health and lifestyle habits and to attend a clinical examination. A total of 7182 adolescents (78% response rate) responded to the questionnaire and clinical examination data were obtained on 6795 adolescents (74% of invitations). In 2005–2008, when the examinees were 19–22 years old, a subsample of 874 cohort members were invited to magnetic resonance imaging (MRI)-scans of the lumbar spine. The subsample comprised those living within a 100 km radius of the city of Oulu. A total of 558 (64% of those invited) representative^27^ individuals attended baseline MRI at a mean age of 21.3. In 2015–2018, at the age of 29–32, those from the subsample who had undergone the baseline lumbar MRI scan were invited to a follow-up MRI study. A total of 375 representative^21^ individuals (43% of those who were originally invited to MRI at baseline) underwent the follow-up scan at a mean age of 30.6 years.

The study followed the principles of the Declaration of Helsinki with voluntary participation. The data were analyzed and handled in a pseudonymized format. The personal details of individual examinees were replaced by identification codes. We adhered to relevant guidelines and regulations in all experiments. Informed consent was obtained from all participants and the research was approved by the Ethics Committee of the Northern Ostrobothnia Hospital District.

### Magnetic resonance imaging of the lumbar paraspinal muscles

The MRI scans of the lumbar spine were performed using 1.5-Tesla imaging. The scanners were Signa HDxt (General Electric, Milwaukee, Wisconsin, USA) in 2005–2008 (baseline) and Optima MR450w (General Electric, Milwaukee, Wisconsin, USA) in 2015–2018 (follow-up). Imaging followed a routine lumbar spine protocol, including T1- and T2-weighted fast-recovery fast spin-echo images in sagittal and transverse planes (repetition time 3960 ms, echo time 116 ms, echo train length 29, number of excitations 4, acquisition matrix 448 × 224 px, field of view 280 × 280 mm, slice thickness 4 mm, and interslice gap 1 mm)^27^. Our institution follows weekly quality assurance protocol for MRI scanners, including measurements for geometric accuracy, and this protocol was in place during both the baseline and follow-up scanning periods.

### Measurements

#### Measurements of paraspinal muscle cross-sectional area

Measurements were performed using Neaview Radiology software version 2.31 (Neagen Oy, Oulu, Finland). Muscle CSA was measured manually using the Neaview specific area measurement tool. We used either T1- (longitudinal relaxation time) or T2- (transverse relaxation time)-weighted images to obtain the optimal image resolution and quality.

**Figure 1:**
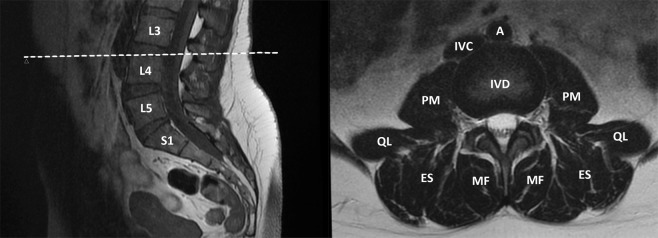
Left: Sagittal plane presenting measurement level. L3 = 3^rd^ lumbar vertebra, L4 = 4^th^ lumbar vertebra, L5 = 5^th^ lumbar vertebra, S1 = 1^st^ sacral vertebra. Right: Axial plane on L4 upper endplate level. MF = multifidus, ES = Erector spinae, QL = quadratus lumborum, PM = Psoas major, IVD = intervertebral disc, IVC = Inferior vena cava, A = Abdominal aorta.

Measurement level was adjusted to the lumbar vertebra 4 (L4) superior endplate level from which the axial scans were projected (Fig. 1). The measurement level was selected in accordance with previous literature^7^ and offered the most optimal visualization of the measurement outlines. If required, the plane was then adjusted three-dimensionally using the Neaview 3D-feature, to match the L4 superior plane. The quality of some of the images was suboptimal and fascia lines had to be approximated. If the muscle outlines of the MRI scans could not be traced from the L4 cranial endplate level, we excluded the measurements.

**Figure 2:**
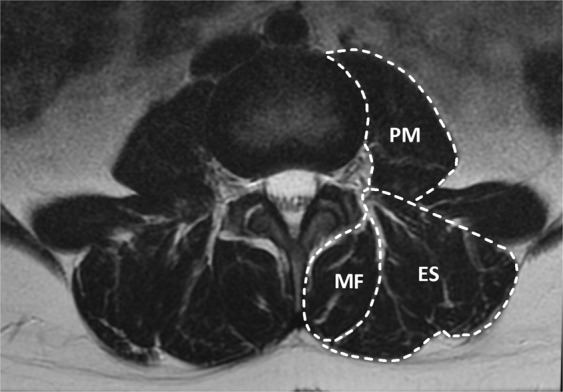
Measurement outlines in axial plane at L4-vertebra cranial endplate level.

The measurements were performed by one researcher (TM) so that the scans of one examinee were measured successively. The reason for sequential measurements was to optimize the measurement level, which was critical for the comparability of the CSA. In the measurements, we distinguished the three separate muscle groups as their own entities: the multifidus (spinotransverse muscle group), the erector spinae and the psoas major (Fig. 2). For further analysis, the CSA of all the above-mentioned muscle groups were added up to form the total muscle area (TMA). The TMA variable was considered to give a more comprehensive view of lumbar spinal musculature and thus supplement the muscle-specific variables. The quadratus lumborum was not included in the measurements because it was not included in the scanning range for all of the patients.

The anatomical borders of MF, ES and PM are described in more detail in the previous literature^17,18^ and were measured accordingly. If the muscle group was not sufficiently measurable from the image, we excluded the measurement. This was the case in some images of the erector spinae, which did not meet the requirements (n = 10) because a major part of the muscle was outside the image boundaries. In other muscle groups (multifidus and psoas major), all the images met the criteria.

#### Reliability of paraspinal muscle CSA measurements

The reliability of the measurements was estimated by randomly choosing 100 (50 baseline and 50 follow-up) MRI scans for remeasurement, which was done by the original researcher, blinded to all earlier measurements. The reassessed scans were measured using the exact same protocol as that used in the original measurements.

### Statistical analysis

We conducted the statistical analyses using the SPSS statistics program (IBM, version 24, 64-bit edition). P values of < 0.05 were regarded as statistically significant. The means and standard deviations of the paraspinal muscle CSA were calculated at baseline and follow-up for both sexes. We further calculated the change in paraspinal muscle CSA for different muscle groups (multifidi, erector spinae, psoas major) for both sides separately, as well as for all muscle groups with both sides combined. The outcomes of these calculations are presented in the results.

To assess measurement error and reliability, we analysed the original and repeated measurements by calculating the intra-rater ICC (intraclass correlation coefficient) and TEM (technical error of measurement) in accordance with the previous literature^21,28,29^. The conclusions of the ICC (two-way mixed model with absolute agreement type for single measures) and TEM calculations are presented in the results.

We used generalized estimating equation (GEE) modelling to analyse the longitudinal data. This regression-based model has been introduced as a suitable analysis method for repeatedly measured data^30,31^. The statistical significance of the age-related change in paraspinal muscle CSA and sex interaction over the follow-up period were tested through GEE. The results of the GEE models with beta (β) estimates, P values and 95% confidence intervals are presented in the results.

## Results

### Study sample

The general characteristics of the study sample are presented in Table 1. The sample size was 298, of which 156 (52.3%) were men and 142 (47.7%) women. The baseline MRI scans were conducted at the mean age of 21.3 (SD = 0.61 yrs), and follow-up scans at the mean age of 30.6 (SD = 0.55 yrs). The average interval between MRI scans was 9.4 years (SD = 0.73 yrs).

**Table 1:** General characteristics of study sample (n = 275–285 due to missing background data).

Characteristic	Men	Women	All
	Mean (SD)	Mean (SD)	Mean (SD)
**MRI characteristics**			
Age at baseline (yrs)	21.2 (0.64)	21.3 (0.58)	21.3 (0.61)
Age at follow-up (yrs)	30.5 (0.58)	30.8 (0.48)	30.6 (0.55)
MRI interval (yrs)	9.3 (0.74)	9.5 (0.70)	9.4 (0.73)
**Anthropometry at age 16**			
Body height (cm)	175.1 (6.8)	163.9 (5.9)	169.9 (8.5)
Body weight (kg)	65.9 (12.1)	55.8 (8.6)	61.3 (11.7)
BMI (kg/m^2^)	21.4 (3.3)	20.7 (2.9)	21.1 (3.1)

### Size and change in paraspinal muscle CSA

The means and standard deviations in paraspinal muscle CSA, and the change between baseline and follow-up over the 10-year follow-up period are presented in Table 2.

**Table 2:** CSA = Cross-sectional area, MFL = Left Multifidus, MFR = Right Multifidus, ESL = Left Erector Spinae, ESR = Right Erector Spinae, PSML = Left Psoas Major, PSMR = Right Psoas Major, TMA = Total Muscle Area.

Variable	Men (n = 156)				Women (n = 142)			
	Age 20	Age 30	Change	%	Age 20	Age 30	Change	%
MFL CSA (mm^2^)	692.8 (131.3)	734.3 (139.2)	+41.5	+6.0	494.1 (93.8)	546.9 (106.0)	+52.8	+10.7
MFR CSA (mm^2^)	721.6 (133.1)	760.2 (142.2)	+38.6	+5.3	510.9 (96.5)	563.4 (104.6)	+52.5	+10.3
ESL CSA (mm^2^)	2188.5 (336.2)	2254.9 (365.2)	+66.4	+3.0	1492.8 (239.8)	1621.0 (257.4)	+128.2	+8.6
ESR CSA (mm^2^)	2142.9 (330.6)	2191.8 (368.1)	+48.9	+2.3	1449.4 (222.0)	1591.7 (257.6)	+142.3	+9.8
PSML CSA (mm^2^)	1794.5 (343.5)	1707.6 (308.5)	−86.9	−4.8	986.1 (176.5)	982.0 (192.3)	−4.1	−0.4
PSMR CSA (mm^2^)	1806.0 (318.3)	1747.4 (306.1)	−58.6	−3.2	1000.6 (174.1)	1014.7 (189.8)	+14.1	+1.4
TMA (mm^2^)	9332.0 (1235.0)	9381.0 (1214.6)	+49.0	+0.5	5935.5 (762.0)	6320.5 (838.3)	+385.0	+6.5

#### Measurement error and reliability

Table 3 shows the results of the Intra-rater ICC (intraclass correlation coefficient) and TEM (technical error of measurement).

**Table 3:** Measures of accuracy (i.e. TEM) and reliability (i.e. ICC) of MRI measurements (n = 50 repeated measurements).

Variable	TEM		ICC	
	Absolute (mm^2^)	Relative (%)	Coefficient	95% CI
MFL20	24.8	4.3	0.972	0.951–0.984
MFL30	22.0	3.5	0.978	0.962–0.987
MFR20	23.7	3.8	0.977	0.959–0.987
MFR30	24.2	3.7	0.974	0.955–0.985
ESL20	78.0	4.3	0.967	0.942–0.981
ESL30	67.5	3.5	0.977	0.956–0.988
ESR20	84.8	4.8	0.961	0.933–0.978
ESR30	58.3	3.1	0.981	0.966–0.989
PSML20	49.7	3.6	0.988	0.979–0.993
PSML30	44.0	3.3	0.989	0.980–0.994
PSMR20	51.0	3.6	0.988	0.978–0.993
PSMR30	40.7	2.9	0.992	0.985–0.995

TEM = Technical error of measurement, ICC = Intra-class correlation, CI = Confidence interval, MFL20 = Left multifidus at age 20, MFL30 = Left multifidus at age 30, MFR20 = Right multifidus at age 20, MFR30 = Right multifidus at age 30, ESL20 = Left erector spinae at age 20, ESL30 = Left erector spinae at age 30, ESR20 = Right erector spinae at age 20, ESR30 = Right erector spinae at age 30, PSML20 = Left psoas major at age 20, PSML30 = Left psoas major at age 30, PSMR20 = Right psoas major at age 20, PSMR30 = Right psoas major at age 30.

According to the intra-evaluator TEM calculations, the mean error in the measurements varied between 2.9% and 4.8%. TEM was higher in the baseline measurements. The ICC coefficients varied between 0.961 and 0.992.

#### Longitudinal assessment of paraspinal muscle CSA changes

Table 4 shows the results of the GEE models (β estimates, 95% confidence intervals and P values respectively).

**Table 4:** Results of GEE models.

Variable	Men (n = 156)		Women (n = 142)		Sex interaction
	β (95% CI)	p	β (95% CI)	p	p
MF (mm^2^)	80.0 (60.4–99.6)	<0.001	105.4 (88.7–122.0)	<0.001	0.053
ES (mm^2^)	115.3 (46.6–184.1)	0.001	270.5 (219.9–321.1)	<0.001	<0.001
PM (mm^2^)	−145.5 (−204.9; −86.1)	<0.001	10.0 (−23.0; 43.1)	0.553	<0.001
TMA (mm^2^)	49.0 (−71.4; 169.4)	0.425	385.0 (301.6; 468.3)	<0.001	<0.001

MF = Multifidus, ES = Erector spinae, PM = Psoas major, TMA = Total muscle area, β = beta estimate, CI = confidence interval.

According to the GEE models, the CSA of MF increased significantly among both sexes (men + 5.7%, p < 0.001; women + 10.5%, p < 0.001). The increase of MF CSA was larger among the women although the difference did not quite reach statistical significance (sex interaction p = 0.053). In addition, the CSA of the erector spinae increased during the follow-up period and the change was significant among both sexes (men + 2.7%, p = 0.001; women + 9.2%, p < 0.001). The increase in the erector spinae was also more notable among the women (p < 0.001). Among the men, the CSA of the psoas major decreased during the follow-up period (−4.0%, p < 0.001). Among the women, the CSA of the psoas major increased slightly, but the change was statistically insignificant (+0.5%, p = 0.553). Total muscle area increased during the follow-up period among both sexes (men 0.5% and women + 6.5%). However, the change was significant only among the women (women p < 0.001; men p = 0.425). According to sex interaction, the CSAs of the ES and TMA increased more significantly among the women (p < 0.001) and the CSA of the psoas major decreased more significantly among the men (p < 0.001).

## Discussion

This longitudinal MRI study aimed to investigate the size and change of lumbar paraspinal muscle CSA at the L4 vertebra cranial endplate-level in early adulthood between 20 and 30 years. Our main findings were that the CSA of the multifidus and erector spinae increased among both sexes and the total muscle area increased among the women but not among the men. Interestingly the increase in MF and ES seemed more distinct among the women. The CSA of the psoas major decreased among the men but not among the women.

The mean CSA of all the paraspinal muscle groups were larger among the men than among the women both at baseline and follow-up, which is in line with the previous literature^7,12^. However, it seems that overall, the paraspinal muscle CSA only increased among the women. One reason for this difference may be the decrease of the psoas major CSA among the men. The CSA of PM showed a decreasing tendency on both sides during follow-up period only among the men.

The underlying reasons for the women’s more significant paraspinal CSA increase remain unclear. Muscle dynamics are multifactorial in nature and muscle size and mass are influenced by the balance between protein synthesis and the degradation process, which in turn is influenced by nutritional factors, hormonal status, injuries, diseases and physical activity^11,32^. Birth weight seems to positively correlate with muscle strength^33^ and lean muscle mass in adult life^34^. Previous literature has suggested that peak muscle mass seems to be attained around the third decade^24^. Muscle mass in relation to body weight seems to start decreasing in the third decade, yet absolute muscle mass seems to be preserved until the fifth decade. This suggests that muscle composition changes to include more fat^24,25,35^. Thus, the increase of intramuscular fat content among women would be a plausible explanation for this difference in CSA increments. However, it is unclear whether this is likely to occur so early in life. In addition, pregnancies and childbirths among women might explain this difference for some extent, as gestational weight gain is a known physiological phenomenon^36^. Unfortunately, the data did not include information on previous pregnancies.

It is also unclear why the CSA of the PM decreased specifically among the men but not among the women. There are known metabolic and endocrinological differences between sexes^11,24,25,35^. Men experience greater losses in muscle mass during ageing but, instead of atrophy, muscle quality seems to decline among older women due to increased fatty infiltration^35^.

Among both sexes, the CSA of the multifidi increased on both sides and the CSA of the left multifidus was larger than that on the right. Also among both sexes, the CSA of the erector spinae increased on both sides, and the CSA of the left erector spinae was larger than that on the right. As mentioned above, the CSA of the psoas major showed a decreasing tendency on both sides during the follow-up period among the men.

Paraspinal muscle morphology and size have been of particular interest in studies investigating low back pain (LBP). Still, how lumbar muscle characteristics is an explaining factor for LBP is far from explicit^1,2,37–42^. A great need for longitudinal studies has been addressed to evaluate causality in this matter^1,39^. Thus, the findings of this study aim to provide prospective insight into outcome measures that may be relevant to the development and management of low back pain, which has a substantial burden of disease.

MRI seems to offer the most optimal modality for assessing muscle properties^17,43–45^. However, validation studies of different modalities are scarce^17,44^. The L4 cranial endplate level was selected as the plane of measurements in accordance with previous literature^7^. In addition, new methods and standardized procedures for assessing muscle fat composition^17,46,47^ and electrodiagnostics^48^ are emerging.

The main strengths of our study are its longitudinal follow-up design, population-based cohort material, relatively large sample size, and reliable muscle measurements. To the authors’ knowledge, only a few previous studies have been conducted in longitudinal settings to assess the paraspinal CSA of the general population^9^ and among patients with LBP^1^. Thus, this study offers new information on the changes in lumbar paraspinal muscle CSA during early adulthood between 20 and 30 years of age.

The main limitations of this study are its use of single-level measurements and its lack of evaluation of muscle composition. The use of single-level measurement as a proxy for paraspinal muscle CSA and fatty infiltration has been criticized, and multilevel evaluation has been suggested^49^. However, this seems to be the case in cross-sectional settings, as paraspinal muscle size and fat infiltration vary at different spinal levels at different points of time. In this study, thanks to our longitudinal dataset, we were able to measure the individual change in paraspinal muscle CSA. Importantly, we took the measurements using corresponding planes from the baseline and follow-up scans. Through GEE, each individual’s paraspinal muscle CSAs at baseline and follow-up were then analysed in a coupled manner. Thus, each individual was essentially compared to themselves, which minimized the inaccuracy associated with using a single measurement plane. The importance of the evaluation of all muscle morphology aspects, principally composition, has been considered in the previous literature^17,50–54^, but for simplicity, our study focused on muscle CSA only. Future studies should evaluate different aspects of muscle morphology and composition at different spinal levels and time points.

Potential sources of error in our measurements were image quality and the limited number of slices in the MRI scans. In the majority of the measurements we utilized T2-weighted images. However, in some of the MR scans, T1-weighted images seemed to offer a more optimal visualization of the muscle outlines. The proportion of intra-muscular fat tissue was not taken into account when CSA of the muscle was measured. However, despite these potential limitations, our intra-rater ICC and TEM calculations demonstrated high repeatability and reliability of measurements and are in line with the previous literature^55^.

In summary, this study assessed the changes in paraspinal CSA in the Northern Finnish population of young adults from 20 to 30 years of age in a longitudinal dataset using repeated MRI scans. We found that the CSA of the multifidus and erector spinae tended to increase among both sexes and that the total muscle area seemed to increase among women but not among men. Considering the wide range of anthropometric variables and muscle morphology perspectives, the results of this study provide many angles for future research, such as the evaluation of sex-specific differences in muscle dynamics, and towards a better understanding of the development of LBP.
